# Molecular mechanism of edema formation in nephrotic syndrome: therapeutic implications

**DOI:** 10.1007/s00467-007-0521-3

**Published:** 2007-06-07

**Authors:** Alain Doucet, Guillaume Favre, Georges Deschênes

**Affiliations:** 1 Laboratoire de Physiologie et Génomique Rénales, CNRS/UPMC UMR 7134, Institut des Cordeliers, 15 rue de l’Ecole de Médecine, 75270 Paris, France; 2 Service de Néphrologie Pédiatrique, Hôpital Robert Debré, Paris, France

**Keywords:** Sodium retention, Collecting duct, Na,K-ATPase, Epithelial sodium channels (ENaCs), Aldosterone, Capillary hydraulic conductivity, Diuretics

## Abstract

Sodium retention and edema are common features of nephrotic syndrome that are classically attributed to hypovolemia and activation of the renin–angiotensin–aldosterone system. However, numbers of clinical and experimental findings argue against this underfill theory. In this review we analyze data from the literature in both nephrotic patients and experimental models of nephrotic syndrome that converge to demonstrate that sodium retention is not related to the renin–angiotensin–aldosterone status and that fluid leakage from capillary to the interstitium does not result from an imbalance of Starling forces, but from changes of the intrinsic properties of the capillary endothelial filtration barrier. We also discuss how most recent findings on the cellular and molecular mechanisms of sodium retention has allowed the development of an efficient treatment of edema in nephrotic patients.

## Introduction

Interstitial edema is a common clinical feature of nephrotic syndrome (NS). It is often massive (up to 30% of body weight) and constitutes a functional constraint, owing to locomotive restriction and eyelid shutting. Expansion of the interstitial compartment volume results from the combination of abnormal renal sodium retention and alterations of fluid transfer across capillary walls.

## Renal retention of sodium in nephrotic syndrome

### Site of sodium retention

Most of our current knowledge on the site and mechanism of renal sodium retention in NS comes from experimental models of the disease, in particular the puromycin aminonucleoside (PAN) rat model. Following a single injection of PAN, rats develop massive proteinuria within 4–5 days and sodium retention within 2–3 days. Sodium excretion resumes after 9–10 days, while proteinuria lasts for 2–3 weeks [1, 2]. Unilateral NS can be induced by the injection of PAN into one of the renal arteries [3]. Early in vivo micropuncture studies in rats with unilateral PAN nephrosis demonstrated that sodium retention in the nephrotic kidney originates beyond the last nephron segment accessible to micropuncture, i.e., between the late distal convoluted tubule and the tip of the collecting duct [3]. This part of the nephron encompasses the connecting tubule and the cortical and outer medullary collecting ducts, which reabsorb sodium and are the major sites of the adjustment of sodium balance under the homeostatic control of aldosterone, and the inner medullary collecting duct, which is able to secrete an overload of sodium in response to atrial natriuretic peptide [4]. Methods allowing in vitro analysis of isolated sub-segments of the distal nephron demonstrated a marked stimulation of sodium reabsorption in the cortical collecting duct (CCD) of PAN nephrotic rats [1, 5]. This stimulation of sodium transport likely extends upstream to the connecting duct but not downstream to the outer medullary collecting duct. In addition, in NS, the inner medullary collecting duct becomes insensitive to the natriuretic action of atrial natriuretic peptide [6–8], thereby preventing any compensation of sodium retention in upstream nephron segments.

### Cellular mechanism of sodium retention in the collecting duct

**Fig. 1:**
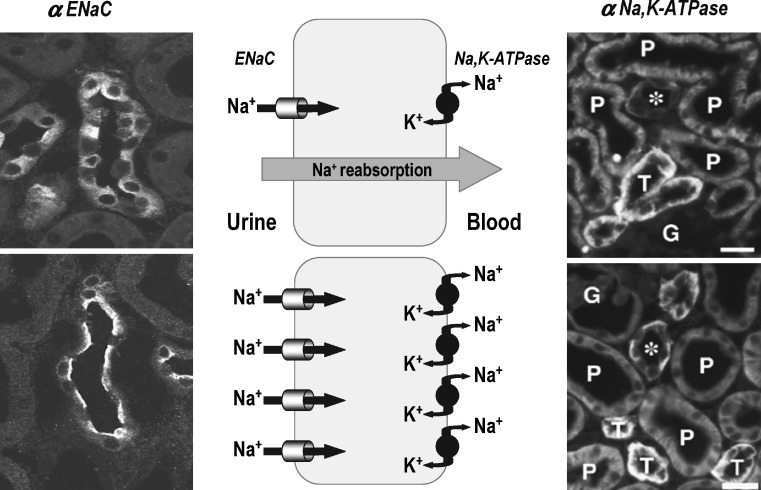
Cellular mechanism of sodium reabsorption in principal cells of collecting ducts from normal rats and nephrotic rats. Sodium reabsorption in principal cells proceeds along a two-step mechanism that includes active extrusion of intracellular sodium ions by the basolateral Na,K-ATPase and passive apical entry of sodium via the amiloride-sensitive epithelial sodium channel (*ENaC*). In CCDs from normal rats (*top panels*), most ENaCs are sequestered in the intracellular compartment of principal cells (*left panel*), and basolateral expression of Na,K-ATPase in collecting ducts (*asterisk*) principal cells is very weak, in comparison with that in thick ascending limbs (*T*) and even proximal tubules (*P*) (*right panel*). Accordingly, the rate of sodium reabsorption is very low. In CCDs from PAN nephrotic rats (*bottom panels*), ENaC is expressed at the apical border of principal cells (*left panel*), and expression of basolateral Na,K-ATPase is drastically increased in collecting ducts (*asterisk*). Polarized increases in expression of ENaC and Na,K-ATPase in principal cells account for increased sodium reabsorption in CCDs. In both normal and nephrotic rats expression of Na,K-ATPase is undetectable in the glomerulus (*G*); in CCDs, unlabeled cells for both ENaC and Na,K-ATPase are intercalated cells (redrawn from [13, 14])

In vitro microperfusion of isolated CCDs confirmed that the rate of sodium reabsorption and the trans-epithelial voltage are negligible in CCDs from control rats, whereas both are high in CCDs from PAN nephrotic rats [5]. CCDs are made of principal and intercalated cells, which account for sodium and water reabsorption and potassium secretion for principal cells, and proton, bicarbonate and likely chloride transport for intercalated cells [4]. In principal cells, sodium reabsorption proceeds along a two-step mechanism (Fig. 1): Na,K-ATPase, exclusively present in the basolateral membrane, energizes the active extrusion of sodium at the expense of ATP hydrolysis. The electrochemical gradient for sodium generated by this primary process drives passive entry of sodium through the apical membrane, which contains selective sodium channels (epithelial sodium channels, ENaCs). Both Na,K-ATPase and ENaCs are targets for multiple, and often coordinated, regulations in CCDs [4].

Increased sodium reabsorption along the CCDs of PAN nephrotic rats, as well as sodium retention and edema formation, are associated with stimulation of both basolateral Na,K-ATPase [1, 9, 10] and apical ENaC [11–13] (Fig. 1), but the latter is dispensable (see below). Stimulation of Na,KATPase is fully accounted for by transcriptional induction of its α and β subunits and targeting of newly synthesized pumps to the basolateral membrane of principal cells [14]. In contrast, stimulation of ENaC mainly results from the targeting of a pre-existing pool of latent intracellular channels to the apical membrane of principal cells and slightly from transcriptional induction of the α and β subunits [11–13]. The patch-clamp technique shows that the intrinsic properties of ENaC (open probability and unitary conductance) are not altered in the CCD of nephrotic animals [13].

### Aldosterone and activation of mineralo-corticoid receptors are not involved in sodium retention in nephrotic syndrome

Historically, it is acknowledged that sodium retention and edema formation result from hypovolemia-induced stimulation of the renin–angiotensin–aldosterone system. Hypovolemia is supposed to proceed as follows: proteinuria induces hypoalbuminemia and reduces plasma oncotic pressure, which generates an imbalance of Starling’s forces across capillary walls, leading to interstitial leakage of fluid and decreased efficient volume [15]. Although this mechanism is consistent with the renal site of sodium retention, with the activation of ENaC and the induction of Na,K-ATPase in the collecting duct, and with the hyperaldosteronemia observed in PAN nephrotic rats [13], a large body of clinical evidence argues against this theory.

- Analbuminemic patients display no sodium retention and no, or only modest, edema, despite low plasma oncotic pressure [16].
- In children with steroid-sensitive minimal change disease, natriuresis resumes at the same time as proteinuria stops, before normalization of albuminemia [17].
- Blood volume is not correlated with plasma oncotic pressure in nephrotic patients [18].
- Among children with relapse idiopathic nephrotic syndrome, (a) 21% display hypertension but only 4% exhibit collapse [19], and (b) only 1% display low blood volume, whereas 17% present hypervolemia [20].
- Intravenous injection of albumin induces volume expansion but promotes only mild natriuresis (reviewed in [21]).
- Blockade of mineralo-corticoid receptor or inhibition of angiotensin-converting enzyme has no effect on natriuresis in most patients [22, 23].

The lack of a role for hyperaldosteronemia in sodium retention in NS was directly demonstrated in PAN nephrotic rats. In this model it is possible to blunt PAN-induced hyperaldosteronemia through bilateral adrenalectomy and corticosteroid replacement through implanted mini-pumps delivering constant physiological level of aldosterone and glucocorticoids. Administration of PAN to these corticosteroid-clamped rats reduces sodium excretion, establishes sodium balance and promotes ascites formation with similar time course and intensity as in adrenal-intact rats [11, 13].

These findings also exclude a possible role in sodium retention of promiscuous activation of the mineralo-corticoid receptor (MR) by glucocorticoid brought about by decreased 11β-hydroxysteroid dehydrogenase type 2 (11β-HSD2). Indeed, use of dexamethasone instead of corticosterone for glucocorticoid replacement prevents MR activation, even in the presence of 11β-HSD2 inhibition. The lack of involvement of MR activation in sodium retention in nephrotic syndrome is consistent with the fact that nephrotic syndrome does not induce potassium secretion.

### None of the known factors that control sodium reabsorption in the collecting duct accounts for sodium retention in PAN-nephrotic rats

Since aldosterone, the major factor that controls sodium reabsorption in collecting ducts, is not involved in sodium retention, several hormones or paracrine factors that modulate this process have been considered as putative candidates:

- Vasopressin (AVP), through activation of its V2 receptors coupled to the cAMP pathway, increases sodium reabsorption in the collecting duct synergistically with aldosterone [24, 25].
- Angiotensin II (AII) increases sodium reabsorption directly through activation of AT1 receptors coupled to phospholipase C, independently of the induction of aldosterone release [26].
- Insulin-like growth factor I (IGF-1) used in the treatment of insulin resistance in type 2 diabetes can induce sodium retention and edema [27], likely through its stimulatory action on sodium transport along the collecting duct [28].
- In mouse collecting duct cells, tumor necrosis factor alpha (TNFα) increases membrane expression of Na,K-ATPase [29], and administration of etanercept, a TNFα receptor antagonist, to diabetic rats induces natriuresis [30].
- More recently, the thiazolidinedione agonists of peroxisome proliferator-activated receptor γ (PPARγ) were reported to induce sodium retention [31] through a stimulatory effect on the collecting duct [32].
- Quite unexpectedly, inhibition of nitric oxide synthase promotes sodium excretion in cirrhotic rats with ascites [33].

**Fig. 2:**
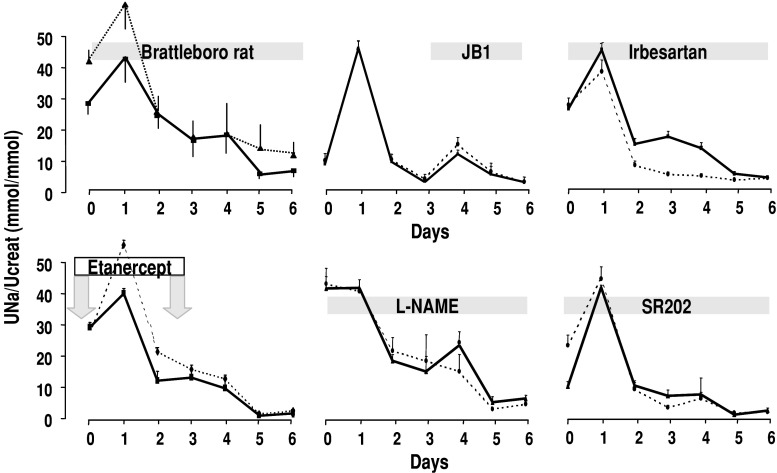
Profile of sodium excretion in PAN nephrotic rats. Daily urinary sodium excretion, expressed as a function of urinary creatinine excretion, following administration of puromycin aminonucleoside (150 mg/kg body wt, intravenously) in normal rats (*dotted lines*) or genetically modified or pharmacologically treated rats (*solid lines*). *Arrows* or *grey boxes* show the time of treatment. Brattleboro rats genetically lack vasopressin secretion. JB1, an inhibitor of IGF-1 receptors, was continuously administered via subcutaneous mini-pumps at a dose of 12 μg/100 g body wt per day, starting on day 3. The antagonist of AT1 receptor, irbesartan, was administered per os at a dose of 2 mg/100g body wt per day. Etanercept, a chimeric antibody directed against TNF receptor, was administered twice (days −1 and 2) at a dose of 0.2 mg/100g body wt. The inhibitor of nitric oxide synthase, L-NAME, was administered twice daily by gavage (0.5 mg/100 g body wt per 12 h) throughout the study. The antagonist of PPARγ, SR202, was given per os at a dose of 20 mg/100 g body wt. All controls were treated in parallel with the vehicle. Values are means ± SE from 4–5 rats

Except for AII, all maneuvers aimed at blocking these different pathways had no effect on sodium retention in nephrotic syndrome (Fig. 2). Only the AT1 blocker irbesartan was able to improve sodium excretion, although not up to normal level. However, this effect was limited to the initial sodium retention observed 2–4 days after PAN administration and did not alter the main effect observed at days 5 and 6. This suggests that AII, independently of aldosterone, may be involved in the early retention of sodium observed after the peak of sodium excretion at day 1.

As a matter of fact, sodium retention in PAN nephrosis unlikely results from the presence of an abnormal blood concentration of any factor, since only the treated kidney displays increased sodium reabsorption in unilateral PAN-nephrotic rats. Rather, alterations in collecting duct function may be accounted for either by a direct effect of PAN on CCD or by the abnormal presence of an unidentified stimulatory factor in the luminal fluid that reaches the distal nephron. The presence of this factor may result from its abnormal filtration secondarily to the alteration of the glomerular filtration barrier (e.g., a high molecular weight protein), or from its generation in the proximal tubule secondarily to alteration of protein/peptide handling.

### Na,K-ATPase is the primary target of sodium retention in PAN nephrosis

Analysis of corticosteroid-clamped rats not only demonstrated that hyperaldosteronemia is not involved in the etiology of edemas in NS, but it also provided insights into the primary molecular target of sodium retention in the CCD. Indeed, in CCDs from corticosteroid-clamped PAN rats, Na,K-ATPase is induced as in adrenal-intact nephrotic rats [13]. In contrast, targeting of ENaC to the apical membrane is fully blunted [11, 13], and the residual channels expressed at the apical membrane are drastically inhibited, as their open probability is reduced >10-fold [13].

Thus, induction of Na,K-ATPase is primarily responsible for sodium retention in PAN nephrotic syndrome. This conclusion likely applies to all experimental models of nephrotic syndrome and, possibly, to the disease in humans, since there is a significant reverse correlation between Na,K-ATPase activity in collecting duct and urinary sodium excretion during the phase of sodium retention in rats with nephrotic syndrome induced by PAN, Adriamycin or mercury chloride [1].

## Edema formation in nephrotic syndrome

Most sodium-retaining states are associated with high blood pressure but not with development of edema or ascites. Edema formation in NS results from the asymmetry of extracellular volume expansion brought about by sodium retention: the vascular volume is not, or only slightly, modified, whereas water and solutes accumulate in the interstitium. Distribution of fluid between vascular and interstitium compartments is governed by fluid exchanges across the capillary wall and by lymphatic draining. Capillary filtration capacity is increased almost twofold in nephrotic patients [34]. This fluid leakage is governed by several parameters according to Starling’s law:

$$ {\text{J}}_{{\text{v}}}  = {\text{L}}_{{\text{p}}}  \times {\text{S}} \times {\left[ {{\left( {{\text{P}}_{{\text{c}}}  - {\text{P}}_{{\text{i}}} } \right)} - \sigma  \times {\left( {\Pi _{{\text{c}}}  - \Pi _{{\text{i}}} } \right)}} \right]} $$

where J_v_ is the trans-capillary flux of fluid, L_p_ the hydraulic conductivity of capillaries, S the exchange surface, P_c_ and P_i_ the hydrostatic pressure of the capillary and interstitium, respectively, σ the reflection coefficient of proteins across the capillary wall, and Π_c_ and Π_i_ the oncotic pressure in the capillaries and interstitium, respectively.

Unexpectedly, data from the literature indicate that the increase in J_v_ in NS is not accounted for by change in the oncotic pressure gradients, as intuitively thought, but by changes in the intrinsic properties of the capillary walls that govern their hydraulic conductance and reflexion coefficient for proteins. This means that the capillary wall is a direct target in NS, to the same right as the glomerular filtration barrier.

### The trans-capillary gradient of oncotic pressure (Π_c_–Π_i_) is unchanged in NS

The lack of edema and ascites in analbuminemic rats and patients [16, 35] has questioned the importance of low plasma oncotic pressure in the genesis of edema in NS. As a matter of fact, the trans-capillary gradient of oncotic pressure is unchanged in analbuminemic rats, owing to a parallel decrease in plasma and interstitium oncotic pressure [35]. Experiments in dogs, in which plasma oncotic pressure was progressively decreased by 50% through plasmapheresis, showed that the interstitium oncotic pressure decreased in parallel and that the trans-capillary gradient remained unaffected. Extracellular volume increased transiently during the phase of variation of plasma oncotic pressure, in response to hyperaldosteronemia and renal sodium retention, but returned to normal level during the period of stabilized low plasma oncotic pressure [36].

Essentially similar observations were made in humans. During NS, it was also observed that the oncotic pressure of the interstitium decreases in parallel with that of the vascular compartment, so that the trans-capillary gradient is only slightly reduced [37]. In addition, diuretic treatments [37] or extracorporeal ultrafiltration [38] allow the withdrawal of significant amounts of edema without significant change in the trans-capillary gradient of oncotic pressure.

Thus, decrease in plasma oncotic pressure in animal models as well as in nephrotic patients does not alter significantly the trans-capillary gradient of oncotic pressure and is neither a determinant parameter in the genesis of edema nor a resistance factor to edema withdrawal.

### The trans-capillary gradient of hydrostatic pressure (P_c_–P_i_) is unchanged in NS

Capillary pressure is unchanged in nephrotic patients [34]. Because soft tissues display an almost infinite compliance, their interstitium pressure increases by only 2 mmHg with their fluid filling [39]. In nephrotic patients interstitial pressure in edematous and non-edematous sectors differs by <4 mmHg [40]. Thus, it can be admitted that the trans-capillary gradient of hydrostatic pressure is not significantly altered in soft edematous tissues during NS.

### The capillary hydraulic conductivity L_p_ is increased in NS

The threshold of venous pressure triggering fluid transfer across capillaries is significantly reduced in NS [34], suggesting that endothelial L_p_ is decreased. The main determinants of the hydraulic conductivity of capillaries are the occlusive junctions (constituted by occludin, claudins and proteins ZO) and adhesive junctions (made of cadherins, catenins and actinin) between endothelial cells. These proteins are direct targets for intracellular signaling cascades, in particular protein kinase C (PKC), which phosphorylates occludin [41] and alters the endothelial permeability [42]. In NS this pathway may be activated through two mechanisms. Firstly, hypoalbuminemia increases L_p_ via an increase in intracellular calcium [43]. Secondly, high plasma levels of TNFα observed in patients with minimal change disease [44] activate PKC and increase L_p_ [45].

### The reflexion coefficient of proteins (σ) is increased in NS

Increased coefficient of reflexion of macromolecules in NS is evidenced by the higher rate of leakage of technetium-labeled albumin towards the interstitium in nephrotic patients than in controls [46]. Because this change in reflexion coefficient is observed in patients with NS of different origin, it is likely not accounted for by the circulating permeability factor of lymphocyte origin responsible for glomerular hyperfiltration in minimal change disease. Increased σ was also reported in blood–peritoneal barrier permeability in PAN-nephrotic rats [47].

## Therapeutic implications

Nephrotic edema results from the combination of renal sodium retention and increased capillary permeability. Although treatment of either of these two alterations would prevent edema, treatment of capillary permeability alone would lead to hypertension. Thus, treatments of edema must primarily target renal sodium retention.

Different therapeutic strategies are needed to prevent edema formation in children with chronic proteinuria and to treat massive edema. In the first case it is sufficient to prevent sodium accumulation, either through limiting dietary sodium uptake or by natriuretic drugs that target the collecting duct, e.g., amiloride. In clinical practice the daily uptake of sodium is usually limited to 0.5 mmol/kg. In PAN-nephrotic rats administration of amiloride starting before the onset of sodium retention fully prevents sodium retention and formation of ascites [5].

More classically, clinicians are confronted with patients who already display massive edema. Treatment aims both at limiting further sodium retention and at promoting the excretion of the mass of sodium and water sequestered in edema. While amiloride is well suited to block further sodium retention, it is inefficient in promoting massive sodium excretion, because, under normal conditions, sodium reabsorption along the collecting duct is quantitatively low. Recourse to more potent diuretics, such as loop diuretics, is restricted by the functional resistance of nephrotic patients to the natriuretic effect of furosemide. Several explanations have been proposed to account for this resistance, but none has been confirmed:

- The pharmacokinetics of furosemide urinary elimination is not significantly altered in nephrotic children [48]
- Binding of furosemide to albumin in the tubular fluid does not account for furosemide resistance, since, despite controversial data [49, 50], inhibitors of furosemide–albumin binding do not improve sodium excretion significantly.
- The intrinsic sensitivity of the Na/K/2Cl transporter of the loop of Henle, the molecular target of furosemide, is not altered in nephrotic rats [5].

Based on our present knowledge of the site and cellular mechanism of sodium retention in NS, another explanation of furosemide resistance, and a therapeutic strategy to circumvent it, can be proposed. In non-nephrotic patients, furosemide decreases sodium reabsorption along the thick ascending limb of the loop of Henle, which increases sodium delivery to the distal nephron. Because the sodium reabsorption capacity of the distal nephron is rather limited, only a small fraction of the overload of sodium is reabsorbed, and the major fraction is excreted in the urine, accounting for the natriuretic effect of loop diuretics. Note that following long-term treatment with furosemide, there are adaptations of the distal nephron which increase its sodium reabsorption capacity and, thereby, reduce the natriuretic effect of furosemide. Because nephrotic patients display a huge sodium reabsorption capacity along their connecting and cortical collecting tubules, most of the overload of sodium brought about by furosemide-induced inhibition of transport in the thick ascending limb is reabsorbed, thereby blunting the natriuretic effect. Thus, the apparent resistance of nephrotic patients to loop diuretics can be circumvented by the inhibition of distal sodium reabsorption with amiloride. As a matter of fact, co-administration of furosemide and amiloride to nephrotic children increases urinary sodium excretion, induces a negative sodium balance and promotes complete edema withdrawal within 1 week [21].

## Conclusion

Whatever their etiology, nephrotic syndromes are always associated with renal retention of sodium. Renal sodium retention results from enhanced sodium reabsorption along the connecting and cortical collecting ducts and from blunted responsiveness of medullary collecting ducts to the natriuretic response to atrial natriuretic peptide. Induction of de novo synthesis of Na,K-ATPase is the primary effector of increased sodium reabsorption. It is not accounted for by any circulating factor, in particular aldosterone, known to stimulate sodium reabsorption along the distal nephron. New research strategies will be required to identify the unknown regulatory pathway that is dysregulated in NS.

Sodium retention in NS does not lead to high blood pressure but leads to an asymmetric expansion of the interstitium, while the vascular volume remains unchanged in most patients. This asymmetry of extracellular volume expansion is accounted for by changes in the intrinsic properties of the endothelial capillary barriers, i.e., an increase in its hydraulic conductivity and permeability to proteins, rather than to an imbalance of Starling’s forces.

Thus, the pathophysiology of nephrotic syndrome relies on at least three disorders: a major alteration of the glomerular filtration barrier responsible for proteinuria and hypoalbuminemia, an induction of distal nephron Na,K-ATPase responsible for sodium retention, and alterations in the capillary permeability accounting for the asymmetry of volume expansion. Although causal relationships between these three events have not been formally established, it is assumed that the glomerular defect engenders both the tubular and the capillary alterations. This conclusion is based on the facts that: (a) whatever its origin, alteration of the glomerular filtration barrier always leads to sodium retention and edema formation, i.e., to the tubular and capillary defects, and (b) loss of function mutations of nephrin, which is expressed in the glomerular slit diaphragm but in neither the collecting duct nor capillary endothelial cells, is sufficient to promote proteinuria, sodium retention and edema [51]. Curiously, in PAN-induced nephrosis, sodium retention precedes proteinuria, suggesting that it is not secondary to the glomerular dysfunction. It should be stressed, however, that microproteinuria may appear sooner and concomitantly with sodium retention.
